# Perezhivanie as a source of children’s development: case of emotional development intervention through visual arts

**DOI:** 10.3389/fpsyg.2024.1476973

**Published:** 2025-01-08

**Authors:** Margarita Gavrilova, Dmitry Kornienko

**Affiliations:** Federal Scientific Center of Psychological and Multidisciplinary Research, Moscow, Russia

**Keywords:** cultural-historical theory, perezhivanie, children development, case study, visual arts

## Abstract

Recent theoretical ideas emphasise the non-direct environmental influences on child development. Environmental factors affect child development by the principle of *refraction* rather than by the principle of *reflection*: any environmental condition is refracted in a child’s mind in unique ways depending on his or her actual abilities and personal attitude to the situation. Complexity and multidimensionality of the relationship between environment and psychological prosseses poses a great challenge to the researchers. This paper provides a theoretical analysis and justification of the prospective use of the concept of ‘perezhivanie’, as a tool for theoretical analyses of child development and a practical phenomenon that allows to explore how a child interprets and emotionally relates to certain events or moments in the environment. Perezhivanie is considered as the unit of analysis of consciousness in cultural-historical theory. A case study of an intervention in emotional development through visual arts is presented as an illustration of using the concept of ‘perezhivanie’ to design practical sessions with children and to analyse the way in which their minds work in learning processes. The findings suggest that the study of perezhivanie for theoretical and practical purposes is promising, as this phenomenon can provide rich data on how a child interprets and relates to a particular event or moment in the environment.

## 1 Introduction

The most effective results in and development can be achieved when the cognitive, emotional and personal potential of the learner is engaged simultaneously (Burger, 2015; Habgood and Ainsworth, 2011). Interventions in education in which this principle can be realized are characterized by the special involvement of learners, relying on their individual experience, interests and motivation (Burger, 2015; Veraksa et al., 2022). However, they do not lend themselves well to scientific study because empirical research requires strict separation of the constructs under study and transparent experiments. The results of ‘discrete’ studies are important for refining scientific understanding in each individual field, but they cannot yet be combined to create a holistic approach to child development. All processes occurring in consciousness are not only closely intertwined with each other but are also uniquely embedded in the context of the child’s life, culture, and time. This complexity and multidimensionality of the relationship between environment and development poses a great challenge to researchers. Studying developmental processes in the context of these and other aspects of child-environment interactions is important, even while recognizing that it is rather impossible to develop such a coherent scientific methodology in the foreseeable future. This paper describes an attempt to use one conceptual framework that may bring us closer to solving the above problem. The concept of ‘perezhivanie’ proposed by Vygotsky (2019) as the unit of analysis of consciousness, was applied in a case-study in an intervention focused on emotional development.

### 1.1 Perezhivanie

Cultural-historical theory considers child development as a process of mastering the cultural experience of mankind. According to this approach, as the child grows up, there is a transition from the natural form of consciousness work to the use of cultural means (Bodrova and Leong, 2018). And they are used not as ordinary tools but are interiorized and determine the work of consciousness (Vygotsky, 1998). Perception, memory, thinking and other mental processes are transformed because of the child’s acquisition of cultural values and social experience that existed long before his birth. But it is fundamentally important to note Vygotsky’s particular understanding of the relationship between environment and child development. Vygotsky emphasizes that the environment does not influence the child in a direct way (Vygotsky, 2019):

*The whole point is that in this or that situation, the influence depends not only on the content of the situation itself but also upon how the child understands or makes sense of the situation* (Vygotsky, 2019, *p. 73*).

According to Vygotsky the influence of the environment on child should be seen by the principle of *refraction* rather than by the principle of *reflection.* The concept ‘perezhivanie’ was suggested by Vygotsky to analyse the refraction of environmental conditions in the mind (Vygotsky, 2019, 2021). Perezhivanie’ was understood as a unity of mental processes aimed at understanding, interpreting and comprehending by the child moments of reality and developing an attitude to them. Thus, perezhivanie is a concept that allows us to establish the role and influence of the social environment on child development. The concept of perezhivanie can be used to explain why children react differently to the same environmental conditions; why each child has unique impressions and experiences when participating in the same activity; why educational posters hanging on the wall do not work even though the child sees them every day; and finally, why a child can develop independently of or in spite of unfavorable conditions. When using perezhivanie as a theoretical tool for analyzing child development, the answer to these questions could be as follows: the environment is a potential source of development that works if the child experiences perezhivanie (Vygotsky, 2019, 2021; Veresov, 2019). Thus, a poster on the wall will remain a colored spot until it becomes the focus of the child’s attention, is explored and comprehended (Vysotskaya et al., 2024). Moreover, even the simplest aspects of the environment are refracted in the consciousness in an individual way. And how a child ‘sees’ a moment of the environment depends on his/her abilities, experience, interests, motivation and personality.

The concept of perezhivanie is one of the key analytical tools needed to fully apply cultural-historical theory to analyze child development (Vygotsky, 1998; Veresov, 2019). Today, the construct of perezhivanie is being conceptualized at the theoretical level to develop a single most complete understanding of it in the context of cultural-historical theory, operationalization and development of diagnostic tools (Cong-Lem, 2022; Fleer et al., 2017; González Rey, 2016; Veresov and Fleer, 2016; Veresov, 2016a,b; Sukhikh et al., 2022). A slow development of the concept of perezhivanie is caused by various reasons, from the incompleteness of cultural-historical theory to inaccuracies in translating the original texts into other languages. Having no equivalent, perezhivanie was translated into English as ‘experience’, which led to conceptual confusion (Veresov, 2017). However, experience, as understood by Vygotsky, is a mirror-like reflection of the environment, whereas perezhivanie involves refracting it through a unique prism of understanding and individual meaning (Smagorinsky, 2011).

There are concerns about the use of the category perezhivanie in empirical research. It is noted that it is a complex and comprehensive construct that requires deep interpretation (Smagorinsky, 2011; Roth and Jornet, 2016). The variability and subjectivity of such interpretations leads to the fact that it can hardly be operationalized at the level of objective indicators (Roth and Jornet, 2016). On the one hand, the concept of perezhivanie does not yet have a clear meaning to rely on, as pointed out by Smagorinsky (2011). But on the other hand, it is recognized as a useful theoretical construct also for empirically working scholars. Roth & Alfredo note that for the first time this category does not oppose person and environment, but ‘captures the identity of person and environment’ (2016, p. 3).

For more than a decade there has been extensive work on integrating the construct of perezhivanie into research activities and important attempts to apply corpus analysis to achieve a common and most correct understanding of perezhivanie in contemporary English-language work (Cong-Lem, 2022). The most detailed treatment of the concept of perezhivanie is presented in a series of papers by Professor Veresov, a bilingual expert in сultural-historical theory (Filipi et al., 2023; Veresov, 2016a, 2016b, 2017). Working with primary sources (including Vygotsky’s unpublished manuscripts), Veresov endeavors to reconstruct an understanding of perezhivanie based on the place, role and connections of this concept with other concepts, principles and laws of theory (Veresov, 2020). As a result of his extensive theoretical work, he defines perezhivanie in his latest works as follows:

*Perezhivanie is a complex nexus, an alloy of different processes and even personal characteristics of a human being, which includes representation, perception, understanding, subjective interpretations, and conscious awareness* (Veresov, 2020).

This paper offers an example of generating and analyzing perezhivanie in practical work with children, relying on its understanding as a source of development in line with Veresov (2021). An intervention session on emotional learning was developed and conducted with using of visual arts as substrate for generating perezhivanie in children. The following sections present the justification regarding the choice of emotional development and visual arts as a goal and means of intervention, together with an outline and analysis of the case study.

### 1.2 Emotional development as a target of intervention

Between the ages of 3 and 11, children actively learn to recognize, name, and regulate emotions and understand the reasons causes them (Pons and Harris, 2005). Emotional development occurs in social interaction because of children’s direct experience of emotions and observation of others’ emotions (Colliver and Veraksa, 2022; Darling-Churchill and Lippman, 2016; Johnstone et al., 2022). There are also targeted emotional learning interventions (Murano et al., 2020). But most often they combine social and emotional target skills with a predominant focus on the former. In a recent systematic review of 19 social and emotional learning interventions, none of the post intervention findings directly relate to emotional development (Murano et al., 2020). Almost all the improvements are related to positive social interaction and reduced behavioral problems, which are not directly related to emotional development. One likely reason for this bias towards children acquiring social skills and improving behavior may be the lack of real-life experience of living and observing emotions in situations close to everyday life in these interventions. The importance of children’s direct experience is also indicated by the results of training sessions where parents were able to change their behavior after participating in the training and significant changes in children’s ability to understand emotions were recorded (Bjørk et al., 2022). In addition, numerous studies showing the important role of intelligence in emotion understanding provide evidence of the need for internal cognitive work to make sense of and analyze emotional experience (Albanese et al., 2010; De Stasio et al., 2014). The significant role of other mental processes in emotional development has also been documented, including memory, cognitive regulation, imagination, and speech (Veraksa and Veraksa, 2023).

Thus, the results of research in the field of emotional development indicate that it is largely a natural process that occurs as a result of a child’s personal emotional experience and observation of others. But this experience does not directly influence the ability to understand the nature and causes of emotions but are individually refracted in the child’s mind. These findings may suggest that a reliance on perezhivanie might be valuable in enhancing the effectiveness of developmental interventions through encouraging reflection on the child’s experience, involving elements of memory, speech, thinking and imagination.

### 1.3 Visual arts as a substance for perezhivanie

Visual arts, like art in general, is a way of (re)constructing life (Larrain and Haye, 2020; Rosin, 2023). Vygotsky proposes to consider art as a set of aesthetic signs aimed at arousing emotions in people. The mission of art is to evoke subjective emotional and cognitive experiences in people, leading to inner transformation and release. Therefore, Vygotsky suggests that perezhivanie should be considered as the unit of art’s influence on consciousness. This means that a work of art ‘works’ for an individual only if he or she experiences a perezhivanie. The issue here is not the hedonistic value of sensory stimuli (aesthetic pleasure), but the work of all mental functions and the state of special emotional sensitivity aimed at perception, understanding, interpretation, emotional response and reflection. Because of its potential to influence consciousness, art has been widely used as a medium of education and psychotherapy for children, adolescents and adults in practice and research (Murano et al., 2020; Varnell, 2022; Wethington et al., 2008; Dolgikh et al., 2022).

Visual arts, among various art forms, occupy a special place in psychological and educational practice. There are isolated examples of using visual arts to promote speech [e.g., facilitating oral language (Chang and Cress, 2014) or thinking (Winner et al., 2006)]. But mostly this art form is effectively used to develop emotional competence (Fancourt et al., 2019; Ebert et al., 2015; Kastner et al., 2021) and reduce stress (Lopes-Júnior et al., 2016; Rew et al., 2014). According to these studies, visual arts is an effective way to develop children’s recognition of emotions, emotional expressions, and empathic understanding of other people’s actions. The following case study presents an attempt to utilize the potential of visual arts for children’s emotional development by drawing on the concept of perezhivanie.

## 2 Case of emotional development intervention through visual arts

### 2.1 Participants

Two siblings participated in the case study: a girl M. (5.3 y.o.) and a boy U. (7.11 y.o.). The children have lived in the same family since birth and are immersed in a common cultural context, residing in Kabardino-Balkaria (Caucasian republic in Russia). Parenting practices in this region are specific compared to the central part of Russia (Bukhalenkova et al., 2021). The most striking features of upbringing relate to the emotional socialization of children, which is reflected in the expectation of more masculine behavior from boys since childhood. Another peculiarity of family upbringing is encouraging children if they help with household chores. Yet in the interview, parents reported that they strive for a secular upbringing, explaining to children that they do not necessarily have to rigidly follow traditional cultural attitudes.

### 2.2 Procedure

**Figure 1:**
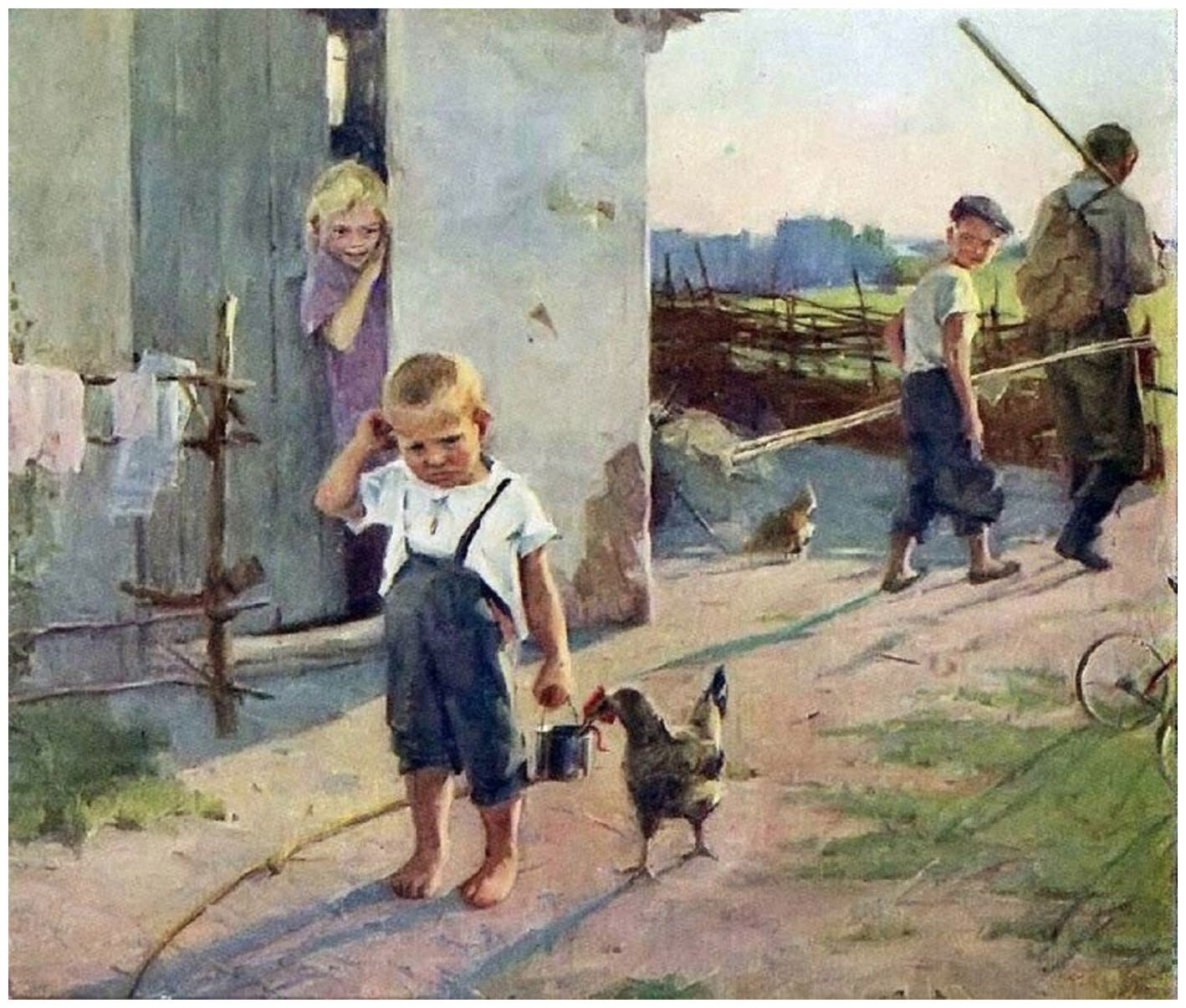
‘Not taken fishing’ (Uspenskaya-Kologrivova, 1955). From the collection of the Tretyakov State Art Gallery.

The described case study is a fragment from the experience of practical work with children aimed at emotional development through viewing, discussing and comprehending visual arts. The case explores the feeling of offence with reference to the painting ‘Not taken fishing’ (Uspenskaya-Kologrivova, 1955) (Figure 1). The full session scenario is presented on OSF platform (https://osf.io/tzdfn/?view_only=7d7d66ed80aa4d02ba79cfc27c210a2f, assessed 06.08.2024).

The sessions were conducted individually with each child in two phases, 1 day apart. At the first meeting, the session itself was conducted with the child, during which the picture was reviewed and discussed. The session was videotaped in order to conduct a detailed analysis of the children’s cues and behavioral displays afterwards. The next day, the child was asked to draw and describe what he/she remembered from the painting and to answer some factual questions (e.g., name the painting).

### 2.3 Key points in the discussion of the painting

The following are the key moments of the teacher’s dialogue with the children. The sessions were one-on-one, but the responses of the two children are presented together for clarity. For ease of analyzing the case study, the eight meaningful blocks of the session are numbered and italicized.

**Table 1:** 

*The teacher helps the child to ‘immerse’ in the picture. Draws attention to the main characters, the composition and the general mood.*	
**Teacher:**	Isn’t it a nice day? It’s sunny, warm. You can run barefoot, or if you do not want to run, you can ride a bicycle. Did you find it? Yes, there it is, the three-wheeled one on the right, but you cannot see it all. Father and his son are leaving the yard. Did you guess where they are going? How did you know? Of course, they are going fishing, there are rods, an oar for the boat, father has a backpack, the boy has a bucket. So it’s serious fishing, not from the shore.
*The teacher draws on the child’s experiences and feelings to evoke his/her own attitude to what is happening in the picture.*	
**Teacher:**	Sit like this, quietly and without distractions, in the boat until you catch some fish for lunch! Do you think it would be easy for you to sit like this, quietly and without moving?
**Girl M. (5.3 y.o):**	It’s hard. I would not do that. But my brother Mark would. He′s always quiet.
**Boy U. (7.11 y.o):**	It would be hard for me.
*The teacher helps the child to understand what is happening in the situation and the relationships of the people depicted.*	
**Teacher:**	Who is the older boy looking back at? Yes, his little brother. Look, do not you think he was getting ready to go fishing too? Why do you think that? Exactly, a bucket of worms in his hand, a homemade fishing rod at his feet. So what happened? The child was so prepared, dreaming of a kind of fish he’d hook! But why does not he follow the others?
**Girl M. (5.3 y.o):**	Maybe he got lost? *(the child does not understand the reason, so the teacher helps to understand that the boy was not taken fishing).*
**Boy U. (7.11 y.o):**	He was in a bad mood. And that’s why he’s not going fishing. *(the child does not understand the reason, so the teacher helps understand that the boy was not taken fishing).*
**Teacher:**	Yeah, so they did not take him fishing! By the way, the painting is called ‘Not taken fishing’. Why did not they take him fishing?
**Girl M. (5.3 y.o):**	I guess fishing’s for grown-ups. He’s just a child.
**Boy U. (7.11 y.o):**	He does not know how. He′s too young.
*The teacher helps the child to understand the relationship between the characters, paying attention to the manifestations of emotions (postures, body language, facial expressions). And refers to the child’s experience, asking him/her to recall situations in which he/she himself/herself has experienced something similar.*	
**Teacher:**	Does the little boy look at those who are leaving? No, he’s turned his back on them. Why?
**Girl M. (5.3 y.o):**	He′s probably wondering where to go. He′s got his back to everyone. Because he’s upset with his brother. And his brother’s got that look on his face. Hm … he’s happy that the younger brother wasn’t taken.
**Boy U. (7.11 y.o):**	No, he’s does not. Because he has a grudge against his father and brother.
**Teacher:**	That’s right, he is upset. How he dreamed of fishing! And it’s all gone. Disappointment! Look carefully at the expression on his face, the tilt of his head. When do you frown like that? When do you lower your head like that?
**Girl M. (5.3 y.o):**	Sometimes. Well … I was this sad when the nanny came to pick me up from the kindergarten and said she was taking me to the hospital.
**Boy U. (7.11 y.o):**	Yeah. When I was a kid, I used to get upset like this.
*The teacher asks the child to repeat the posture and facial expression of the boy through body experience. While the child repeats the pose, the teacher comments emotionally: ‘Just now the boy had a homemade fishing rod in his hand, and suddenly he threw it on the ground and raised his hand to his head: eh, what an injustice!’*	
*The teacher asks additional questions to clarify the intensity of the emotions of the upset child.*	
**Teacher:**	Look, does the child notice that while he is standing like this, the hen is pulling the worm out of his bucket? Why?
**Girl M. (5.3 y.o):**	The boy sees that it wants to drag the worm away. *(the teacher explains).*
**Boy U. (7.11 y.o):**	The boy does not notice the hen pecking the worm. Because he does not care anymore.
**Teacher:**	In fact, the boy has taken so much offence that he cannot see what’s around him.
*The teacher asks additional questions, drawing attention to how the other characters in the picture feel about the upset boy.*	
**Teacher:**	And if he turns around, will he feel that his older sister, who is peering out of the house with curiosity, and his brother sympathize with him? Do you know what it is to sympathize?
**Girl M. (5.3 y.o):**	No, I do not know that word. *(the teacher explains the meaning of the word ‘sympathize’).* No, they do not. Well, the brother might want to take him along. But he’s got a happy face.
**Boy U. (7.11 y.o):**	Hmm … (looking away). I think they sympathize.
*The teacher suggests thinking about possible ways out of the situation (developing strategies for regulating emotions). He refers to the child’s empathy and prosocial skills, asking the child to think of something to say to the hero of the picture to support him.*	
**Teacher:**	Or maybe the mother will appear at the door of the house, come over, stroke his head and say: ‘Do not be upset, baby, do not be offended! Daddy did not promise you anything. Fishing is a long, serious business. Be patient, you’ll grow up a little more, and you’ll be my third fisherman!’. What would you say to the boy to encourage him?
**Girl M. (5.3 y.o):**	I can see the mother here. I’d tell him, ‘Do not be sad, go tell your mum everything.’
**Boy U. (7.11 y.o):**	Be patient. A year or two. You’ll go fishing.

### 2.4 What were the children’s impressions of the painting the next day?

**Figure 2:**
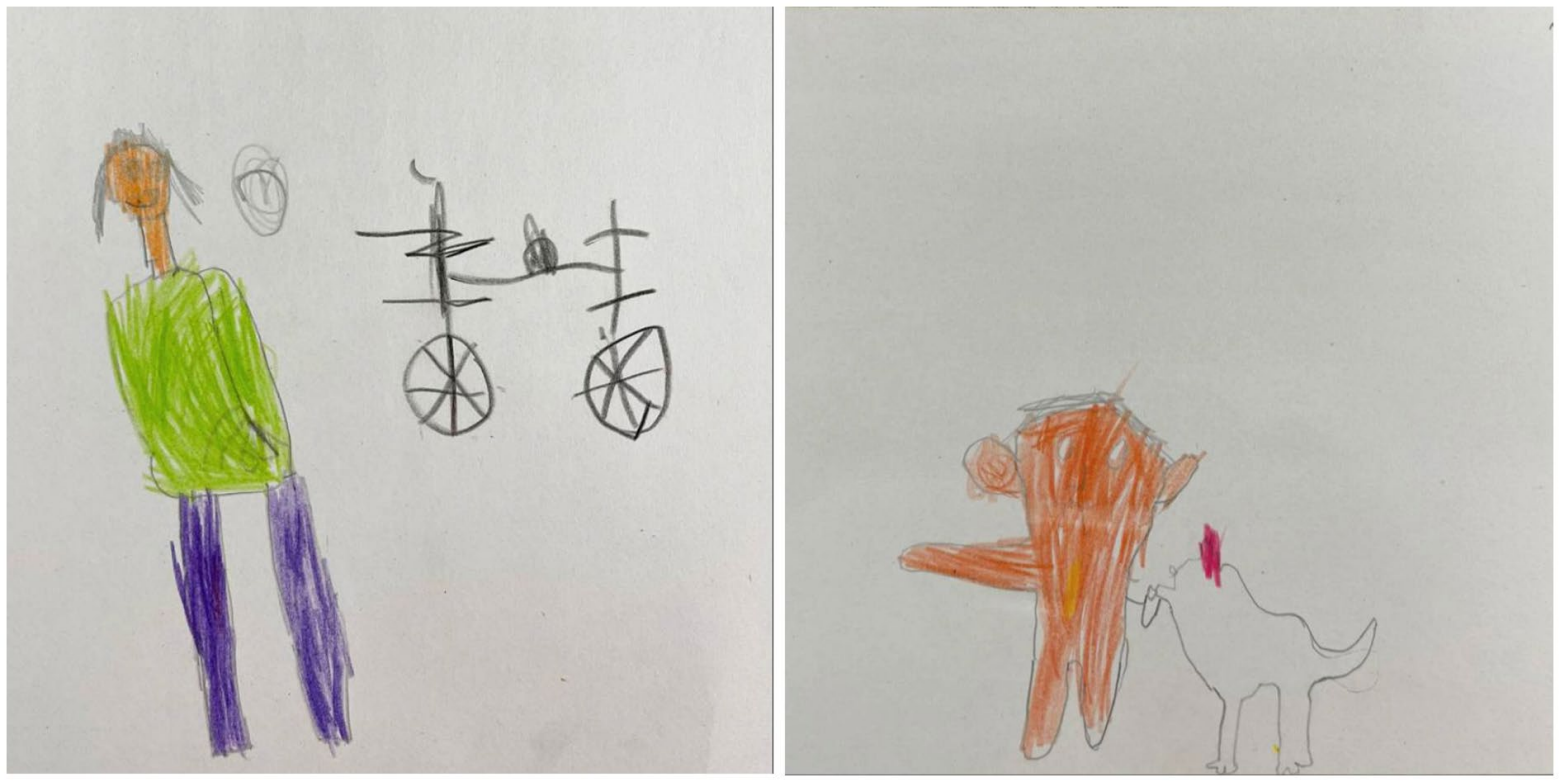
Drawings of children made from memory the day after the session on the picture ‘Not taken fishing’ (Uspenskaya-Kologrivova, 1955). **(A)** Picture by girl M. (5.3 y.o) (Girl M.’s verbal explanation of what is depicted in the drawing: ‘There is a bicycle and a person drawn here. The person is smiling). **(B)** Picture by boy U. (7.11 y.o) (Boy U.’s verbal explanation of what is depicted in the drawing: ‘There is a boy and a hen drawn here. And a bucket. The red one is a worm’).

The next day, the teacher asked each child to draw and tell what they remembered from the picture and answer a few questions. The instructions for the drawing were as follows: ‘Remember yesterday we watched and discussed the painting? Can you please draw what you remember from it?’. The children’s drawings are presented below (Figure 2).

The following are children’s verbal comments on what and why they depicted in their drawings, as well as answers to questions about the content of the picture information and whether they have personal experience of perezhivanie of an emotional state similar to that experienced by the boy in the picture.

**Table 2:** 

*Dialogue with the girl M. (5.3 y.o).*	
**Teacher:**	Tell me, what did you draw?
**Girl M. (5.3 y.o):**	A bicycle and a man are drawn here. The man is smiling.
**Teacher:**	Why do you remember these things in particular?
**Girl M. (5.3 y.o):**	Why? You said to draw what you remembered. I remembered this.
**Teacher:**	And tell me, what was going on in the painting?
**Girl M. (5.3 y.o):**	There was a boy who wanted to go fishing. He wasn’t allowed. He′s just a baby. He prepared some worms. Even a fishing rod. But father took … took … only his older brother. And his older sister laughed at him for not being taken fishing.
**Teacher:**	What was the name of the painting? Do you remember?
**Girl M. (5.3 y.o):**	Yes, ‘Little brother wasn’t taken, but big brother was.’
**Teacher:**	Have you had similar situations like this boy?
**Girl M. (5.3 y.o):** No.	
*Dialogue with the boy U. (7.11 y.o).*	
**Teacher:**	Tell me, what did you draw?
**Boy U. (7.11 y.o):**	There’s a boy and a hen. And a bucket. The red one is a worm.
**Teacher:**	Why do you remember these things in particular?
**Boy U. (7.11 y.o):**	I drew it to … Well, if I had not drawn it … If that moment had not happened, the picture would not have matched. Yesterday’s picture would not match this one. This fragment is the most memorable. You remember it because it’s closer.
**Teacher:**	And tell me, what was going on in the painting?
**Boy U. (7.11 y.o):**	The father and the eldest son went fishing. The younger son wasn’t taken. And the older sister was looking out of the house. The boy had a bucket and a homemade fishing rod. And he threw the rod on the ground because he was upset. And raised his hand to his head. The hen was taking worms from his bucket. And the boy noticed it, but he did not care.
**Teacher:**	What was the name of the painting? Do you remember?
**Boy U. (7.11 y.o):**	Yes. ‘The son wasn’t taken fishing.’
**Teacher:**	Have you had similar situations like this boy?
**Boy U. (7.11 y.o):**	Yeah. I was getting ready to go to the shop. But I did not go. It was today.
**Teacher:**	And did you feel the same way as that boy?
**Boy U. (7.11 y.o):**	No, not the same. Not that much.

## 3 Discussion

The present study provides a theoretical rationale and analyzes the application of perezhivanie as a source of development within a session on children’s emotional development through visual arts. Through a case study aimed at emotional development through the visual arts, empirical observations are obtained on how children interpret and relate to situations in different ways. It was expected that through the analysis of children’s remarks, behavior, drawings and narratives we would be able to observe how information about the studied emotion and its manifestations is individually refracted in children’s minds. Also, based on the understanding of perezhivanie accumulated by Veresov (2021), it was expected that the attempt to generate children’s perezhivanie towards new information may contribute to higher learning efficiency.

Emotional development has been chosen as a target of intervention for several reasons. Firstly, it is one of the areas of child development. A great place in the formation of emotional development is taken by the naturally acquired experience of one’s own emotional perezhivanie, observation of others, and gradual assimilation of culturally accepted ways of expressing and regulating emotions. Secondly, this area yields to purposeful development weaker in comparison with such closest areas as acquisition of social skills or positive behavior. Thirdly, there is theoretical evidence and indirect empirical evidence that emotional development inherently involves perezhivanie. For example, the relationship of emotion understanding, a major component of emotional development, to nonverbal intelligence and other mental abilities (De Stasio et al., 2014) indicates that internal work is required to make sense of emotional experience by engaging an arsenal of mental processes. That is, the experience received by the child does not directly influence emotional development but is refracted in his consciousness depending on the actual abilities. That is why we considered the perezhivanie-based intervention to be the most illustrative and potentially effective in the field of children’s emotional development. Visual arts have been used as a substance for perezhivanie, because a picture selected according to certain criteria not only provides an example of a realistic depiction of an emotional state, but also reflects the complex and always unique context of life, culture and time. It is also important that the purpose of art is to evoke emotions and the experience of inner emotional and cognitive work. It may seem that any depiction of emotion could be used for this purpose, including cartoons, which are more modern and appealing to children. But following Vygotsky’s idea, artistic work, including visual art, is aimed at creating a perezhivanie, which is achieved largely through the combination of cognitive and emotional components and talented artistic execution (Vygotsky, 2019). For these and other reasons, visual arts are widely used as a teaching and psychotherapy tool in practice and research (Murano et al., 2020; Varnell, 2022; Wethington et al., 2008).

The case study explored feelings of offence through viewing, discussing and comprehending the picture ‘Not taken fishing’ (Uspenskaya-Kologrivova, 1955). The session was conducted with two children individually, in two phases with a break on 1 day. On the first day the activity itself was conducted, and on the second day children were asked to draw and tell what they remembered from the picture and answer some factual questions. Two children participated in the case study: girl M. (5.3 y.o) and boy U (7.11 y.o). The children are siblings living in a region with rather vivid cultural specifics. Thus, they are bilinguals growing up in a bicultural environment (more details in the Participants background section). The decision to consider the case of siblings here was made to control for family and wider social environment factors. In doing so, the different ages and genders, along with all the individual characteristics of the children, can have a potential impact on the emergent of perezhivanie in relation to the picture and the session as a whole. A discussion of each case is presented below.

***Case with girl М. (5.3 y.o).*** During the whole session, girl M. kept a positive attitude, smiled and made jokes. For example, after the session, she laughingly said that her mother could have calmed the boy down by telling him: ‘Let them go fishing, and I’ll give you a phone.’ This statement indicates both the girl’s joking attitude and her sincere misunderstanding of the depth of the boy’s offence and confusion at not being taken fishing. In the girl’s picture of the world, fishing is only one alternative thing he could have done. Therefore, her own drawing shows a bicycle as the thing she remembers most from the painting. The bicycle is indeed present in the painting. Obviously, the artist included it in the composition in order to highlight the child’s depicted life drama. But in the girl’s perezhivanie, the bicycle completely removes the existing conflict, so the boy in her drawing is smiling. It is indicative that in her verbal retelling the girl quite accurately reproduces the content of the painting and understands the socio-emotional context of the situation. She independently notes and explains the gloating in the painting of the elder brother and sister, but remains emotionally indifferent to the situation. Interestingly, the teacher’s explanations did not significantly pay off in the girl’s progress of understanding the depth of the boy’s experience of confusion and offence. For example, during the session, the girl demonstrated full understanding of the teacher’s explanation that the boy was ‘so much upset that he does not see what is around him’. But according to the conversation the next day this was not learnt.

***Case with boy U. (7.11 y.o).*** At the beginning of the session boy U. had a positive attitude and was calmly looking at the painting. But as he became immersed in the story, his appearance became concerned and serious. The turning point was the realization that the boy from the picture did not go fishing not because of his bad mood (as it seemed to the child initially), but because he was not taken. After that the child practically stopped smiling, began to look at the painting more often and more attentively. His voice became quieter, and his gaze turned inward. When asked why the boy was not taken fishing, he noted not only his age (like his sister), but also the fact that the boy ‘does not know how to fish yet’. Unlike his sister, he correctly stated the reason why the boy does not look at his father and brother leaving. He noted with particular thoughtfulness that the boy in the painting ‘does not care anymore’ that the hen is pulling out the worm prepared for fishing. Illustratively, this very fragment appears in the boy’s own drawing, which he comments on as follows: ‘If that moment had not happened, the picture would not have matched. This fragment is the most memorable’. In his picture, the worm is a symbol of the unfulfilled desire and intentions of the boy-fisherman, it is one of the key semantic elements of the painting. Thus, in many ways the drama of the painting lies in the fact that, while the boy may not know how to fish, but he himself dug up the worms. At the same time, boy U. does not pay attention to the interpersonal relations reflected in the painting. They diminish for him against the background of the drama of the child-fisherman. Both in the picture and in the verbal story the boy emphasizes the hero’s indifference to what happens around the boy-fisherman after he was not taken, ‘he did not care’. The boy mentioned that he was familiar with the feeling that the boy in the painting was experiencing and recounted a similar situation from his own life. But mentioned that he had experienced similar emotions ‘not as much strong’.

In the session, both children were given explanations about emotions and the relationships between the depicted characters, which could improve the ability to understand emotions and theory of mind. We believe that the effectiveness of these explanations could be related to whether it becomes the child’s perezhivanie: how relevant are the explanations and new information in the context of the child’s personal experience, motivation, emotions? Replicas and behavioral displays during the session, as well as verbal and pictorial representations, indicate that the boy rather than the girl did experience perezhivanie towards the key emotional drama conveyed in the painting. Of the two children, the boy relied on the teacher’s explanations in his drawing and story the next day. It cannot be argued that it is the presence of perezhivanie that could account for the progress in emotion understanding and emotion words as a result of targeted instruction. Perhaps the boy is able to perceive, analyze and remember information better during the learning process due to his older age. Nevertheless, being indifferent to the drama unfolding in the picture, the girl seemed less interested in understanding the teacher’s explanations. Another point worthy of attention in analyzing the cases is the children’s personal experiences and the cultural expectations transmitted to them by their environment. In this context, the situation depicted in the painting will relate differently to the children’s experience. For a girl, going fishing is not a meaningful event and perezhivanie of offence is quite normal. For a boy, the opposite is true. Fishing in the company of other adult men is a welcome event that will emphasize his maturity and masculinity. And offence is an indication of weakness and may be condemned in the existing cultural context.

Based on the analysis of the case study, several theoretical conclusions can be drawn about perezhivanie as a source of emotional development through viewing, discussing and making sense of visual arts. The first, a conversation based on a painting as an art form can be effective in generating a perezhivanie, because, as Vygotsky wrote, the mechanism of building a perezhivanie is at the heart of the aesthetic response. At the same time, of course, the emergence of perezhivanie is not guaranteed. The second, combination of verbal and non-verbal expression of the child’s understanding of the painting is effective because developmental heterochrony may be present at this age. The two forms of representation complement each other and allow a more accurate reconstruction of the child’s perezhivanie. The third, child’s reference to the main symbol of the painting in his or her retelling or drawing may indicate the presence of perezhivanie with the main drama of the painting. Since the symbol most often accumulates the main meaning and emotional content of the work. The fourth, personal experience and cultural expectations transmitted to the child determine the occurrence of perezhivanie in relation to a situation.

The study of perezhivanie as a phenomenon can provide valuable data on how a child interprets and emotionally relates to a particular event or moment in the environment. The observations and conclusions drawn from the case study of emotional development intervention through visual arts described in this paper indicate that practical research on perezhivanie for theoretical and practical purposes is promising, and the use of painting as an art form can be effective in generating perezhivanie.

Based on our theoretical and practical work, we would like to outline several directions for further research into the study of perezhivanie and its role in children’s learning and development. Firstly, according to the observations described above, it seems promising to investigate whether the fact that a child experiences perezhivanie towards the content or form of the picture in question can really influence the effectiveness of purposeful learning. Secondly, it is important to clarify whether the occurrence of the perezhivanie is related to the child’s correlation of new information with his or her own life experience. Thirdly, do the cultural expectations of the child determine what kind of perezhivanie the child will have with the situation at hand?

## Data Availability

The original contributions presented in the study are included in the article/supplementary material, further inquiries can be directed to the corresponding author.
